# Rac1 Targeting Suppresses Human Non-Small Cell Lung Adenocarcinoma Cancer Stem Cell Activity

**DOI:** 10.1371/journal.pone.0016951

**Published:** 2011-02-09

**Authors:** Shailaja Akunuru, Joseph Palumbo, Qihui James Zhai, Yi Zheng

**Affiliations:** 1 Division of Experimental Hematology and Cancer Biology, University of Cincinnati, Cincinnati, Ohio, United States of America; 2 Molecular Developmental Biology Program, University of Cincinnati, Cincinnati, Ohio, United States of America; 3 Division of Hematology and Oncology, Children's Hospital Research Foundation, University of Cincinnati, Cincinnati, Ohio, United States of America; 4 Department of Pathology, University of Cincinnati, Cincinnati, Ohio, United States of America; Universidade de São Paulo, Brazil

## Abstract

The cancer stem cell (CSC) theory predicts that a small fraction of cancer cells possess unique self-renewal activity and mediate tumor initiation and propagation. However, the molecular mechanisms involved in CSC regulation remains unclear, impinging on effective targeting of CSCs in cancer therapy. Here we have investigated the hypothesis that Rac1, a Rho GTPase implicated in cancer cell proliferation and invasion, is critical for tumor initiation and metastasis of human non-small cell lung adenocarcinoma (NSCLA). Rac1 knockdown by shRNA suppressed the tumorigenic activities of human NSCLA cell lines and primary patient NSCLA specimens, including effects on invasion, proliferation, anchorage-independent growth, sphere formation and lung colonization. Isolated side population (SP) cells representing putative CSCs from human NSCLA cells contained elevated levels of Rac1-GTP, enhanced *in vitro* migration, invasion, increased *in vivo* tumor initiating and lung colonizing activities in xenografted mice. However, CSC activity was also detected within the non-SP population, suggesting the importance of therapeutic targeting of all cells within a tumor. Further, pharmacological or shRNA targeting of Rac1 inhibited the tumorigenic activities of both SP and non-SP NSCLA cells. These studies indicate that Rac1 represents a useful target in NSCLA, and its blockade may have therapeutic value in suppressing CSC proliferation and metastasis.

## Introduction

Lung cancer remains the leading cause of cancer deaths worldwide. The disease is broadly classified into two histo-pathological groups - small cell lung cancer (SCLC) and non-small cell lung cancer (NSCLC), with the later group representing ∼80% of lung cancer cases. Adenocarcinomas occur in about 55% of the NSCLC and 40% of all lung cancers. Despite of continued development of cancer therapeutics, currently the overall five year survival rate for lung cancer patients is less than 15%[1]. Often, existing chemotherapy, radiation therapy or surgery can only partly remove the tumor burden, leaving behind therapy resistant cancer cells that may regenerate the tumor at the primary site and/or metastasize to secondary sites to initiate new tumors.

Recent publications have reported the identification of cancer initiating or cancer stem cells (CSCs) from blood [2], brain [3], breast [4], colon [5], [6], hepatic [7], pancreatic [8], prostrate [9], [10], as well as lung cancers [11], [12], [13]. The CSCs are identified either by unique cell properties such as Hoechst dye exclusion (Hoechst dye-low side population) or by expression of specific surface markers such as CD133, ALDH, or CD24/CD44, and they are frequently associated with chemotherapy and radiation therapy resistance. CSCs are defined by their stem cell like self-renewal capabilities, their ability to differentiate into cell types that constitute the bulk of the tumor, and to initiate tumors at a significantly reduced dosage in mouse xenograft studies [7], [14]. NSCLC initiating cells have been isolated from human lung cancer cell lines based on increased Hoechst 33342 dye efflux activity [12]. The Hoechst dye low side population (SP) cells are enriched for tumor initiating activity compared to non-side population (NSP) cells and express elevated ABCG2 and other multi-drug resistance transporters that may mediate therapeutic resistance.

The advancement of the cancer stem cell theory has led to the proposal that targeting CSC's can lead to eradication of the residual therapy resistant tumor cells in patients. Recently Gupta *et al* suggested that inducing differentiation of CSC by using salinomycin, a selective potassium ionophore can block mammary CSC activity and metastasis [15]. However, several recent reports have shown that CSCs and non-CSCs can be plastic and inter-convertible in nature [16]–[17]. For example, JARID1 negative cells were shown to represent a transient slow cycling non-CSC population that can give rise to fast cycling JARID1 positive CSCs in melanoma (13). There is evidence that non-CSCs can convert to CSCs through interaction with extracellular matrix and other environmental cues (14). This raises the possibility that approaches solely targeting CSCs are not sufficient for cancer therapy, because the remaining non-CSCs may be reprogrammed to CSCs to reinitiate tumorigenesis.

Rac1 is an intracellular molecular switch that transduces signals in a variety of oncogenic pathways. It is frequently found to be elevated in expression and/or activity in a variety of tumor cells and regulate important cellular processes relevant to cancer cell behaviors, including gene expression, cell proliferation, actin cytoskeleton remodeling and is essential for cell directional migration and adhesion. Rac1 activity can influence cell cycle progression and survival, and it was shown to be required in K-ras mediated lung tumor growth in a murine spontaneous lung cancer model [18]. However, whether Rac1 contributes to human NSCLA tumor growth and/or metastasis, particularly if Rac1 plays a role in regulating CSCs, requires further investigation. In the current work we show that Rac1 is critically involved in NSCLA cell migration, invasion and lung metastasis of SP cells, therefore serving as a useful therapeutic target by inhibiting tumor initiation and metastasis of the CSC population of NSCLA.

## Materials and Methods

### Cell culture

A549, H23, H1299 and H441 cells were cultured according to the guidelines from ATCC. Human bronchial epithelial cells (HBEC) were generous gift from Dr. Jeffery Whitsett (Cincinnati Children's Hospital Medical Center). Primary patient lung adenocarcinoma samples were obtained with written consent from patients under an approved Institution Review Board protocol by University of Cincinnati Scientific Review Committee (IRB# 01-09-27-07), and were used in the experiments according to Cincinnati Children's Hospital Medical Center Scientific Review Committee (IRB # 07-06-57) that the identity of the patients remains anonymous. Tumors were minced and resuspended in DMEM containing 0.5 mg/ml Liberase (Roche) and 1% penicillin and streptomycin. After 45 minute incubation, slurry of cells was passed through 100 micron filter and total cells were washed, plated in 10% fetal bovine serum containing growth media. Epithelial cancer cells were enriched by growing cells in sphere culture conditions.

A549, H23, H1299, H441, HBEC or primary adenocarcinoma cells were infected with lentivirus expressing YFP tagged scrambled shRNA (scr) or Rac1 shRNA1 or Rac1 shRNA2 described previously [19]. Scrambled shRNA construct was generous gift from Dr. Lee Grimes (Cincinnati Children's Hospital Medical Center) and Rac1 shRNA constructs were generous gift from Dr. Jim Mulloy (Cincinnati Children's Hospital Medical Center). After 72 hours of infection, YFP positive cells were sorted using FACS and utilized for different experiments.

For Rac1 mutant rescue experiments, four mismatch point mutations were made in the shRNA binding sites of Rac1 cDNA in the MIEG3 vector using site-directed mutagenesis kit (Stratagene, Agilent technologies) per manufacturer's directions. A549 cells were infected with Rac1 mutant expressing retrovirus and after 72 hours, GFP positive cells were sorted using FACS. The cells were subsequently infected with lentivirus expressing scr shRNA or Rac1 shRNA. After 72 hours, GFP^+^ YFP^+^ cells were used to perform several functional assays.

### Cell proliferation assays

Cells were plated (2000 cells/well) on 96 well plate in triplicates. Number of live cells on each day was determined by non-radioactive MTS proliferation assay (Promega).

For BrdU incorporation assay, BrdU (10 µg/ml) was added to cells at 60% confluency for 2 hours at 37°C. Cells were collected, fixed, stained and FACS analysis was performed as described previously [20]. To detect BrdU positive cells in CD133^+^ and CD133^−^ populations, cells were stained with CD133/2-APC antibody (Miltenyi Biotechnologies Inc.) after BrdU staining. The BrdU positive cells were gated from both CD133^+^ and CD133^−^ cells.

### Soft agar colony formation assay

Cells were seeded (10,000 cells/well) in 0.3% low melting point agarose made in growth media containing 10% fetal bovine serum and layered on top of 0.6% agarose in growth media. Number of colonies formed after either 2 weeks (A549, H1299) or 3 weeks (H441, H23) was counted under light microscope.

### Sphere formation assay

Cells (10,000 cells/ml) were plated in suspension culture conditions in serum-free sphere media (DMEM:F12 containing 0.4% BSA, 10 µg/ml insulin, 10 ng/ml EGF, 10 ng/ml FGF) on 6-well plates pre-coated with 1% agarose to prevent cell attachment. Media was replaced every 2–3 days and number of spheres formed in 2 weeks was counted under light microscope.

### Adhesion assay

Plates were coated with 50 µg/ml fibronectin overnight at 4°C and blocked with 2% BSA for 2 hrs at 37°C. After blocking, Cells were plated (10,000 cells/well) and incubated at 37°C for 60 minutes. Non-adherent cells were aspirated and plates were washed three times with PBS. Numbers of cells attached to the wells after washes were determined by using non-radioactive proliferation MTS assay.

### Migration and invasion assays

For trans-well migration assay, 50,000 cells were added to upper chamber in serum free media and migration at 37°C towards 10% FBS containing growth media was determined either after 24 hours (A549, H1299, H23) or 48 hrs (H441). Cells migrated through the membrane were fixed, stained with Giemsa stain (Sigma) and counted under light microscope.

For invasion assay, lower chambers of matrigel coated invasion plates were coated with 10 µg/ml fibronectin overnight at 4°C and cells invading through matrigel were fixed and stained either after 48 hours (A549 cells) or 72 hours (H441 cells) similar to migration assay.

### Immuno-staining

Cells were plated on fibronectin coated slides and after 18–20 hours cells were fixed using 3.7% Formaldehyde. Cells were stained for actin cytoskeleton (Rhodamine-Phalloidin, Invitrogen), nuclei (DAPI, Invitrogen) using standard immuno-staining methods described previously [21]. Alternatively cells were stained with either phospho-FAK (Focal Adhesion Kinase, Millipore) or vinculin (Sigma) or phospho-paxillin (Cell signaling Technologies).

### Side-population, CD133 cell staining and isolation

Cells are trypsinized and washed with PBS. Cells are stained with Hoechst 33342 staining buffer as described previously at a final concentration of 5 µg/ml Hoechst 33342 [22] for side population (SP), or with anti-CD133 antibody for CD133 positive cells. The cells were analyzed or sorted for SP/CD133^+^ cells by flowcytometry.

To obtain Rac1 knockdown in SP or CD133^+^ cells, cells were infected with lentivirus expressing YFP tagged scr or Rac1 shRNA and after 72 hours cells were stained for side population or CD133. YFP positive SP or non-SP cells were sorted for either Western analysis or functional assays.

### Rac1-GTP pull-down assay

To perform Rac1 pull down assays, cells were lysed by adding lysis buffer containing 20 mM Tris HCl pH 7.6, 100 mM NaCl, 10 mM MgCl, 1% Triton X-100, 0.2% SDS, protease and phosphatase inhibitors directly to adherent cells. Cell lysates containing equal amounts of protein were incubated with glutathione beads conjugated to GST-PAK1 containing active Rac1 interacting domain and processed further as described previously [23].

### Lung colonization assay

Use of mice as xenograft hosts was approved by the IACUC committee at Cincinnati Children's Hospital Medical Center (Protocol# 8D06052). Specified number of cells were suspended in PBS and injected intravenously into immune compromised NOD/SCID/γc -/- (NSG) mice by tail vein injection. At the end of the study, lungs were fixed in Bouin's solution to count number of tumors.

### Subcutaneous xenograft assay

Specified number of cells are suspended in 200 µl of PBS:matrigel mix (1∶1 volume) and injected subcutaneously into the flanks of immune compromised NOD/SCID/γ-/- (NSG) mice. After 3–4 weeks of injection tumor size was measured weekly using calipers and the tumor volume was determined by using formula 0.52XLXW^2^ cm3.

### Tumor cell homing assay

Cells expressing YFP were injected intravenously into NSG mice. After 24 hours, lungs were isolated after perfusion with PBS. Total lung cells were isolated by Liberase digestion and total cell count was determined by Hemavet cell counter. Percentage of YFP positive cells was determined by flow-cytometric analysis of total lung cells. Homing index was determined by calculating percentage YFP positive cells homed to lung normalized to control cells.

## Results

### Rac1 targeting impairs proliferation and colony formation of human NSCLA cells

**Figure 1:**
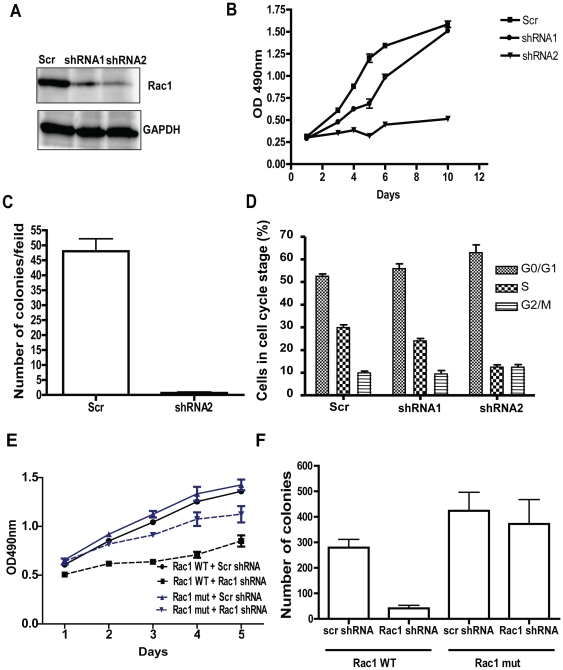
Knocking down Rac1 expression effectively inhibits human non-small cell lung cancer cell proliferation. (A) Cell lysates collected from either scrambled shRNA (Scr) or Rac1 shRNA (shRNA1, shRNA2) A549 cells were subjected to Rac1 western blot analysis. GAPDH was used as loading control. (B) Infected cells were sorted and plated on 96-well plate and proliferation assay was performed using MTS reagent. Assay was performed in triplicates and above is one representative of three independent experiments. (C) Infected cells were sorted, plated for soft agar colony assay and colonies per field were counted after 2 weeks. Assay was performed in triplicates and above is one representative of three independent experiments. (D) Infected cells were sorted and incubated with BrdU in log phase of cell growth. Cells are trypisinized, stained with BrdU antibody, 7AAD and cell cycle analysis was performed using flowcytometry. Assay was performed in triplicates and above is one representative of four independent experiments. Error bars represents SD. (E) Control cells or cells expressing shRNA resistant Rac1 mutant were infected with scr or Rac1 shRNAs, sorted and plated on 96-well plate. Proliferation assay was performed using MTS reagent. Assay was performed in triplicates and above is one representative of two independent experiments. (F) Infected cells were sorted, plated for soft agar colony assay and colonies were counted after 10 days. Assay was performed in triplicates and above is one representative of two independent experiments.

To investigate the role of Rac1 GTPase in NSCLA cell growth, A549, H441, H1299, and H23 cells were infected with lentivirus encoding scr or Rac1 shRNA (shRNA1, 2). Western blot analysis revealed efficient knockdown of Rac1 protein with both shRNA constructs (50% and 90% respectively compared to scr) in A549 cells (Fig. 1A). Rac1shRNA1 expression partially reduced the proliferation of the lung cancer cells while Rac1shRNA2 more potently inhibited cell growth (Fig. 1B) and caused a significant decrease of number of colonies grown in soft agar colony formation assay (Fig. 1C). Further, cell cycle analysis performed by BrdU staining and FACS analysis revealed a decrease in S-phase and a corresponding increase in G0/G1 phase of the cell cycle upon Rac1 targeting (Fig. 1D). In H441 cells, Rac1 shRNA2 led to ∼75% reduction in Rac1 protein (Fig. S1A), and a relative minor effect on proliferation (Fig. S1B). In H1299 and H23 cells, Rac1 knockdown caused a significant reduction in proliferation (Fig. S1C, S1E) and soft agar colony formation (Fig. S1D, S1F). To determine if the Rac1 knockdown effects on proliferation and soft agar growth are specific to Rac1, we performed shRNA-resistant Rac1 cDNA mutant rescue experiments in the knockdown cells. Expressing a Rac1 shRNA resistant mutant cDNA could mostly rescue the proliferation of Rac1 knocked down in A549 cells without a detectable effect on the scr shRNA treated control cells (Fig. 1E). Similarly we observed a rescue of soft agar colony formation by expressing shRNA-resistant Rac1 cDNA mutant (Fig. 1F). Together, these results indicate that Rac1 is required for the proliferative potential of NSCLA cells.

### Rac1 knockdown results in decreased adhesion, migration and invasion of NSCLA cells

**Figure 2:**
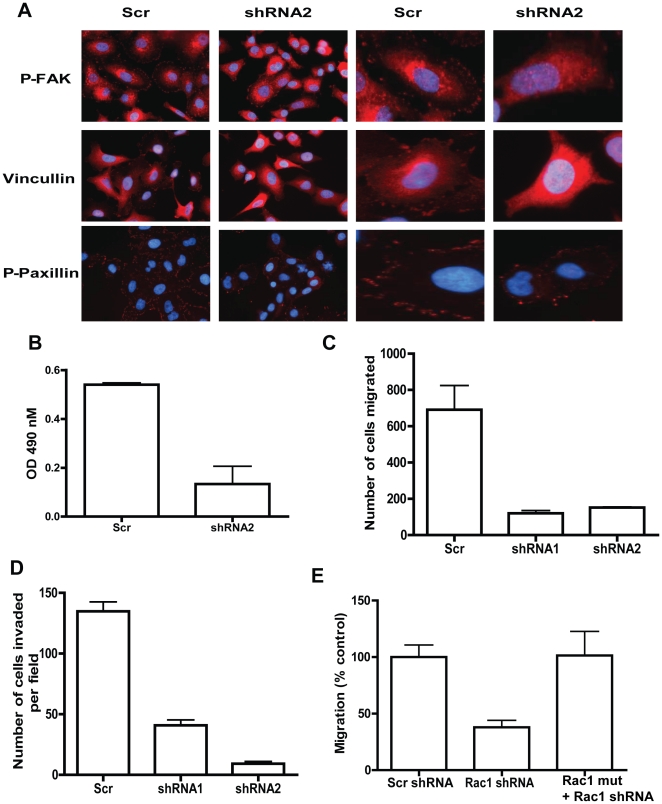
Targeting Rac1 blocks non-small cell lung cancer cell adhesion, migration and invasion. (A) Infected A549 cells were sorted, plated on fibronectin coated slides and stained with either p-FAK (top panel) or vinculin (middle panel) or p-Paxillin (lower panel) and DAPI. Images were collected using fluorescent microscope at 40X magnification. Images above are representative of several images obtained from two independent experiments. (B) A549 sorted cells were plated on fibronectin coated 96-well plate for *in vitro* adhesion assay and cells attached to plate after 1 hour was determined using MTS reagent. Adhesion assay was performed with five replicates and the data is representative of three independent experiments. (C) Sorted cells were plated on trans-well migration plates and migration of cells toward 10% FBS was measured overnight. Assay was performed in replicates and above data was representative of three independent experiments. (D) Sorted A549 cells were plated on matrigel coated invasion plates and migration of cells toward 10% FBS and 10 µg/ml fibronectin was measured after 48 hours. Assay was performed in triplicates and the above is a representative of three independent experiments. Error bars represents SD. (E) Control or cells expressing Rac1shRNA resistant mutant were infected with Rac1 shRNA and sorted. Cells were plated on trans-well migration plates and migration of cells toward 10% FBS was measured overnight. Assay was performed in triplicates and above data was representative of two independent experiments.

Consistent with the known role of Rac1 in cytoskeleton regulation, Rac1 knockdown in both A549 and H441 cells resulted in altered actin cytoskeleton organization (data not shown). Suppression of Rac1 in A549 cells caused decreased focal adhesion complex formation, with control cells exhibiting robust focal adhesion complexes visualized by immunostaining for the focal adhesion proteins p-FAK, vinculin, and p-paxillin whereas the Rac1shRNA2 infected cells demonstrated reduced focal adhesion complex formation (Fig. 2A). We also observed decreased p-FAK, p-paxillin and p-MLC in Rac1 knockdown cells compared to control cells by Western blot analysis (Fig. S2A). Consistent with the decreased adhesion complexes in Rac1shRNA infected cells, adhesion to fibronectin was reduced in these cells compared to control cells (Fig. 2B). Similar to the effect on proliferation, Rac1 partial knockdown in H441 cells resulted in a relatively minor effect on adhesion to fibronectin compared to A549 cells (Fig. S2B). In addition, Rac1 knockdown in both A549 and H441 cells resulted in decreased trans-well migration and invasion activities compared to control cells (Figs. 2C, 2D, & Fig. S2C, Fig. S2D). Similarly, Rac1 knockdown in H1299 or H23 cells drastically decreased migration compared to control cells (Fig. S2E, Fig. S2F). Expressing a shRNA-resistant Rac1 cDNA mutant was able to completely rescue the migration phenotype of Rac1 knockdown in A549 cells (Fig. 2E). These data demonstrate the importance of Rac1 in NSCLA cancer cell adhesion, migration and invasion.

### Rac1 knockdown prevents lung colonization of NSCLA cells in mice

**Figure 3:**
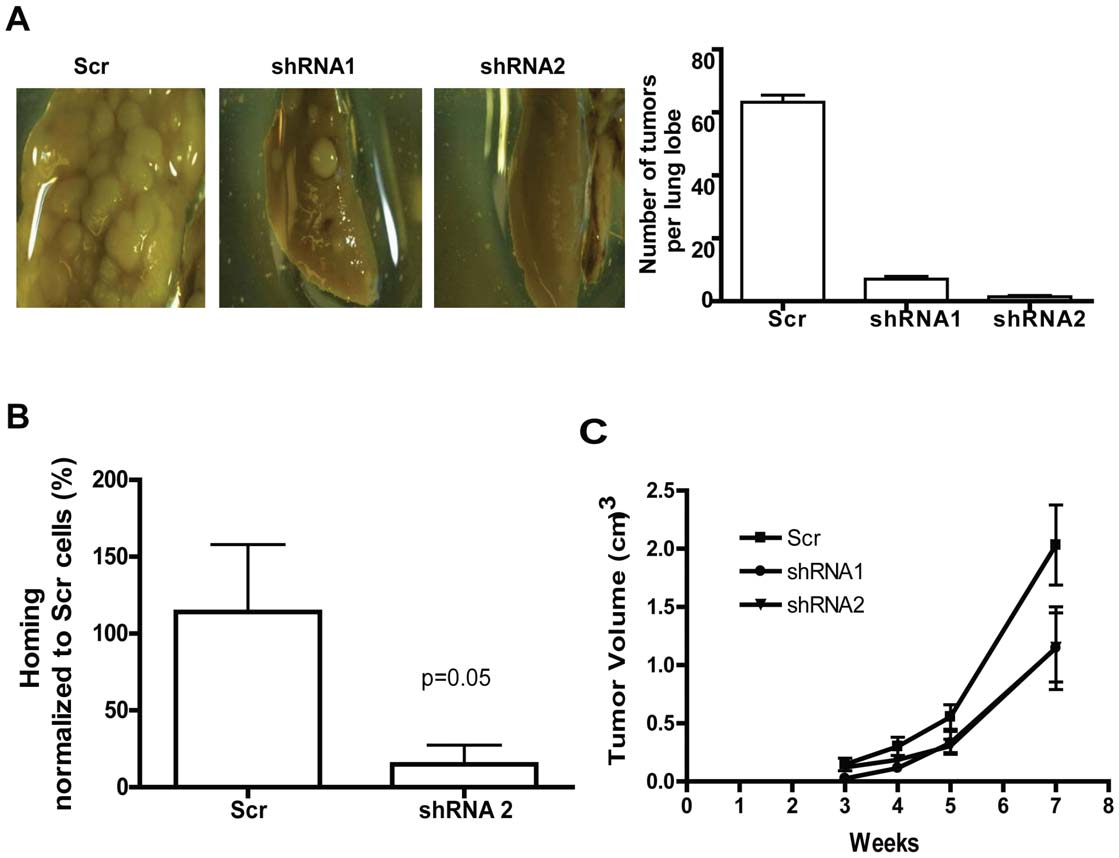
Knocking down Rac1 expression suppresses lung cancer cell homing and tumor growth in the lung of recipient mice. (A) 5×10^5^ A549 cells were injected into tail vein of NSG mice (n = 6 per condition) and lungs were dissected after 8 weeks. Lung were stained with Bouins solution and destained in 70% ethanol. Quantification of lung colonization data was shown in the right panel. Error bar represents SE. Depicted is a representative of two independent experiments. (B) Tumor cell homing assay was performed as described in methods (n = 6 per condition in each experiment). Homing index was measured as percentage of YFP positive cells detected in lung, normalized to control. Depicted is a representative of three independent experiments. (C) 5×10^5^ scr or Rac1 shRNA infected cells were injected subcutaneous into flanks of NOD/SCID mice and tumor volume was measured weekly for 7 weeks. Error bar represents SE.

Next, we examined the effect of Rac1 knockdown on tumorigenesis and metastatic behavior of lung adenocarcinoma cells. Rac1 knockdown or control cells were injected intravenously into immuno-deficient NSG mice in a lung colonization model. Both A549 (Fig. 3A) and H441 cells (Fig. S3A) expressing Scr (500,000 cells/mouse) caused tumor formation in the lungs while Rac1 knockdown cells (500,000 cells/mouse) failed to form tumors in the lungs 8 weeks post-injection. As expected, Rac1shRNA1 infected cells that showed a partial Rac1 protein knockdown formed reduced number of tumors. To determine if the effect on lung colonization is related to a homing defect of the Rac1 knockdown cells in the lung, we tested the ability of the tumor cells to home to the lung tissue 48 hours after tail vein injection. Flow cytometry analysis revealed that the YFP^+^ Rac1 knockdown cells displayed significantly decreased lung homing activity compared to the YFP^+^ Scr cells (Fig. 3B). In addition, subcutaneous xenograft of the tumor cells (500,000 cells/mouse) in NSG mice established that Rac1 knockdown cells had delayed tumor development and reduced tumor volume compared with the control cells (Fig. 3C). Interestingly, sphere formation of H441 cells, which correlates with tumor initiating potential, was also compromised upon Rac1 knockdown (Fig. S3B) whereas no effect was observed by Rac1 knockdown on sphere forming activity of the non-transforming HBEC control cells (Fig. S3C). Thus, it is likely that Rac1 knockdown effects tumor cell lung colonization, growth due to a combined effect on cancer cell homing and proliferation in the lung.

### Side population cells contain elevated Rac1-GTP and increased migration, invasion, and lung colonization activities

**Figure 4:**
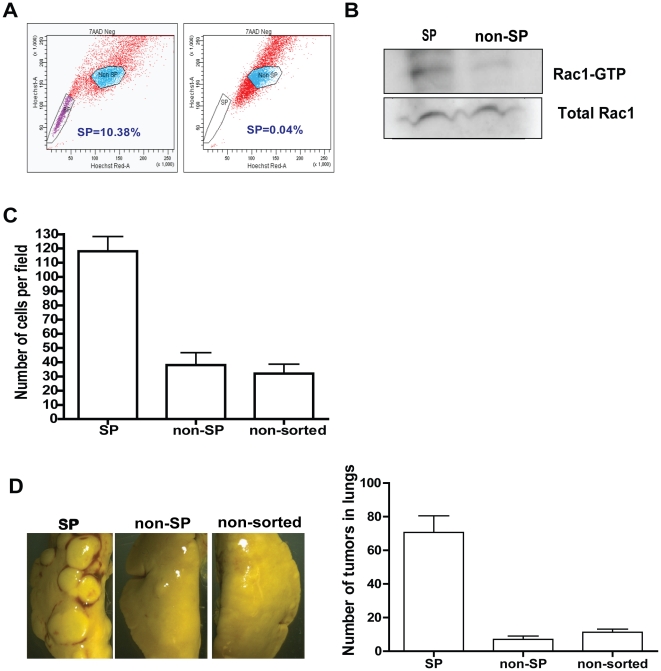
Side population cells possess elevated Rac1 activity, increased migration, invasion and proliferative activities, and enriched tumor initiating activity in mouse lung. (A) A549 cells were stained with Hoechst 33342 dye and analyzed by flowcytometry for side population (left panel). Cells were treated with 10 µM Fumitremorgen for inhibitor control (right panel). Depicted is a representative of several SP analyses. (B) Cell lysates collected from sorted SP and non-SP cells were subjected to GST-PAK pull down assay and processed for Rac1 western blot analysis to determine the Rac1 activity. Total Rac1 blot was used as a control. Depicted is a representative of three independent Rac1 activity pull-down assays. (C) Sorted A549 cells were plated for trans-well migration assay and cells migrated overnight towards 10% FBS were stained and counted. Above is a representative of three independent experiments and error bars represents SD. (D) 5×10^4^ sorted SP and non-SP cells were injected into tail vein of NSG mice (n = 4 per condition). Lungs were dissected out at the end of 12 weeks. Right panel shows quantification of lung colonization data. Error bar represents SE. Above is a representative of three independent experiments.

The cancer stem cell theory suggests that a fraction of the cancer cell population is enriched for tumor initiating capability, thus requiring preferential targeting to achieve therapeutic benefits. Side population is one of the stem cell markers that has been used to isolate lung cancer stem cells [12]. We isolated SP cells by flow cytometry from A549 cells (Fig. 4A) and confirmed by RT-PCR that they express increased ABCG2 transporter (Fig. S4A). Interestingly, SP cells contained increased Rac1 activity (Fig. 4B) and displayed increased migration compared to non-SP cells or parental cells (Fig. 4C). Inhibition of the Rac1 activity by using a small molecule Rac inhibitor, NSC23766, resulted in decreased migration and invasion of SP cells (Fig. S4B, S4C), suggesting elevated Rac1-GTP in the SP cells contributes to these tumor cell behaviors. Furthermore, the SP cells showed increased lung colonization capability *in vivo* compared to non-SP or parental cells (Fig. 4D). Interestingly, despite distinct tumor initiating activities *in vivo*, SP and non-SP cells proliferated at a similar rate as the parental cells *in vitro* (Fig. S4D). However, SP cells displayed increased colony formation activity when growing in soft agar compared to non-SP and parental cells (Fig. S4E). The residual tumorigenic activity exhibited by the non-SP cells was not due to impurity of these cells, because parallel FACS analysis found over 99.9% enrichment for non-SP cells from the Hoechst 33342 dye sorting (data not shown). Therefore it is likely that a plasticity of the non-SP cells allows them to give rise to cancer initiating cells resulting in the observed residual tumorigenicity.

SP cells were also detected in H441 cells at a lower percentage than A549 cells (Fig. S4F; 0.5–2% vs. 4–10%). Similar to A549 SP cells, H441 SP cells displayed increased lung colonization in immune compromised mice compared to non-SP cells (Fig. S4G), and formed more colonies in soft agar compared with non-SP and parental cells (Fig. S4H).

These results lead us to conclude that SP cells from NSCLA represent a subpopulation in the bulk tumor cells that contain elevated Rac1 activity, increased migration, invasion, anchorage-independent growth activities, and are enriched for cells that are capable of colonizing lung. They also suggest that non-SP cells could remain tumorigenic, albeit with reduced CSC activity, to give rise to tumors.

### Rac1 knockdown suppresses adhesion, migration, and invasion of both SP and non-SP cells

**Figure 5:**
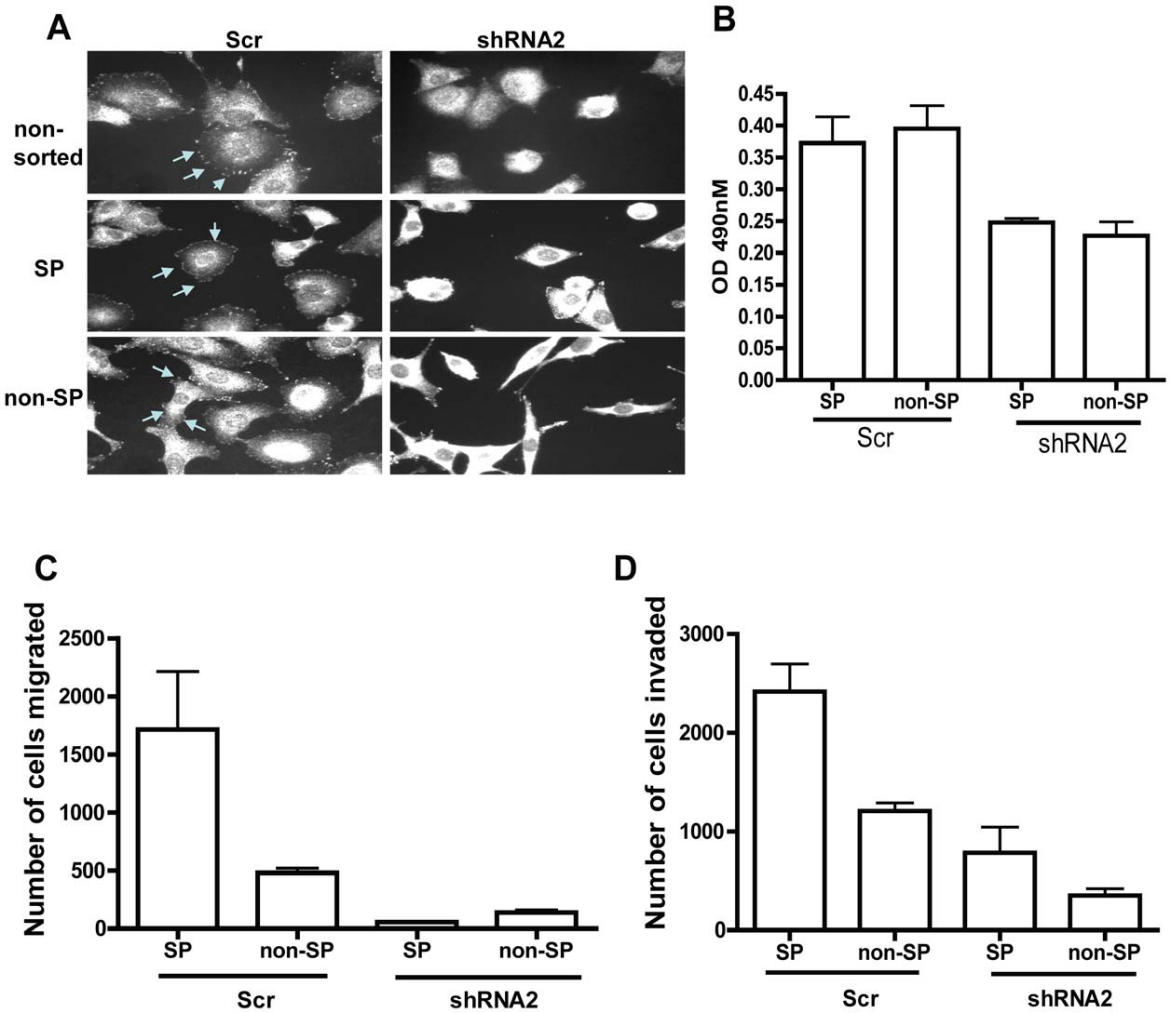
Targeting Rac1 effectively suppresses the adhesion, migration and invasion activities of SP cells as well as non-SP cells. (A) Sorted cells were plated on fibronectin coated slides, fixed and subjected to immunostaining with p-FAK antibody. Cell images were collected at 40X magnification using Fluorescent microscope. Above depicted are representative of multiple images collected. (B, C, D) A549 sorted cells were either plated on fibronectin coated 96-well plate for *in vitro* adhesion assay (B), on trans-well plates for migration assay (C) or matrigel coated invasion plates for invasion assay (D). All assays were performed in triplicates and error bars represent SD. Depicted are representative of three independent experiments.

To further examine the effect of Rac1 knockdown on SP cells, A549 cells were infected with lentivirus either containing scr or Rac1 shRNA, and SP and non-SP cells were isolated by flow cytometry. Western blot analysis confirmed the effectiveness of Rac1 knockdown in both SP and non-SP cells (Fig. S5A). In line with our earlier data on parental cells, Rac1 knockdown altered the cytoskeletal organization of both SP and non-SP cells (Fig. S5B), and reduced focal adhesion complexes as visualized by p-FAK immunostaining of both SP and non-SP cells (Fig. 5A). Consistently, the adhesion activity of both SP and non-SP cells to fibronectin was also decreased (Fig. 5B). Further, Rac1 knockdown decreased migration and invasion of both SP and non-SP cells (Fig. 5C, 5D). Thus, Rac1 targeting can suppress migration and invasion of both SP and non-SP cancer cells.

### Rac1 targeting decreases proliferation and lung colonization of both SP and non-SP cells

**Figure 6:**
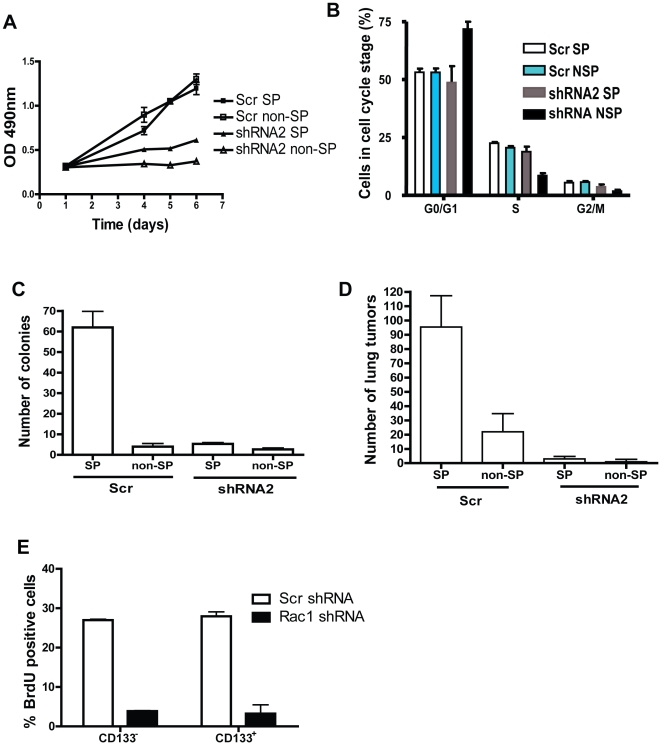
Rac1 knockdown inhibits the proliferation and tumor seeding activities of SP cells as well as non-SP cells *in vitro* and *in vivo*. (A) Sorted cells were plated in 96-well plate and proliferation assay was performed using MTS reagent. Assay was performed in triplicates and error bars represents SD. Depicted is a representative of three independent experiments. (B) A549 sorted cells were incubated with BrdU and cell cycle analysis was performed by BrdU staining and flowcytometric analysis. Assay was performed in triplicates and error bars represent SD. Above is a representative of two independent experiments. (C) Sorted cell were directly plated for soft agar colony formation assay and the number of colonies formed were counted after 2–3 weeks. Assay was performed in triplicates and error bars represent SD. Above is a representative of three independent experiments. (D) 50,000 sorted cells were injected into tail vein of NSG mice and the lungs were isolated for analysis after 12 weeks. Number of lung tumors was counted under light microscope and error bar represents SE. Results are representative of three independent experiments. (E) Cells were infected with either scr or Rac1 shRNA and incubated with BrdU. Cells were stained with CD133 antibody during the BrdU staining described in methods. BrdU positive cells gated from CD133^+^ and CD133^−^ cells were analyzed by FACS.

To examine whether the observed inhibition of proliferation of the overall cancer cell population by Rac1 knockdown is due to a specific effect on CSCs, we next tested the growth properties of isolated SP and non-SP cells before and after transduction of Rac1-specific shRNA. The proliferation of SP and NSP cells *in vitro* appeared similar under standard tissue culture conditions, and Rac1 knockdown blocked *in vitro* proliferation of both SP and non-SP cells to the similar extent (Fig. 6A). BrdU labeling showed that both SP and non-SP cells were inhibited in S-phase transition with a corresponding increase in G0/G1 phase of cell cycle after Rac1 knockdown (Fig. 6B). While scrambled RNA did not affect the increased colony formation activity of SP cells in a soft agar assay compared with non-SP cells in either reduced serum or normal serum conditions (Fig. 6C; data not shown), Rac1 shRNA was able to significantly reduce colony formation of both SP and non-SP A549 cells. In tail-vein injected NSG mice, the lung colonization of Rac1 shRNA SP cells was drastically decreased compared to scr cells and the corresponding non-SP cells showed no tumor colonization activity (Fig. 6D). These results provide strong evidence that Rac1 targeting is effective in inhibiting proliferation and metastasis of both SP and non-SP cells. To test if the Rac1 knockdown effects on proliferation are applicable to cancer stem cells marked by CD133, a BrdU incorporation assay was performed in which BrdU positive cells were gated in CD133^+^ or CD133^−^ population of both scr and Rac1 shRNA treated cells. We observed a decrease in the BrdU^+^ cells in both CD133^+^ and CD133^−^ subpopulations upon Rac1 knockdown (Fig. 6E). Thus, Rac1 is required for proliferation of the CSC population.

### Rac1 knockdown decreases migration, invasion, sphere formation, and metastatic activities of primary human NSCLA cells

**Figure 7:**
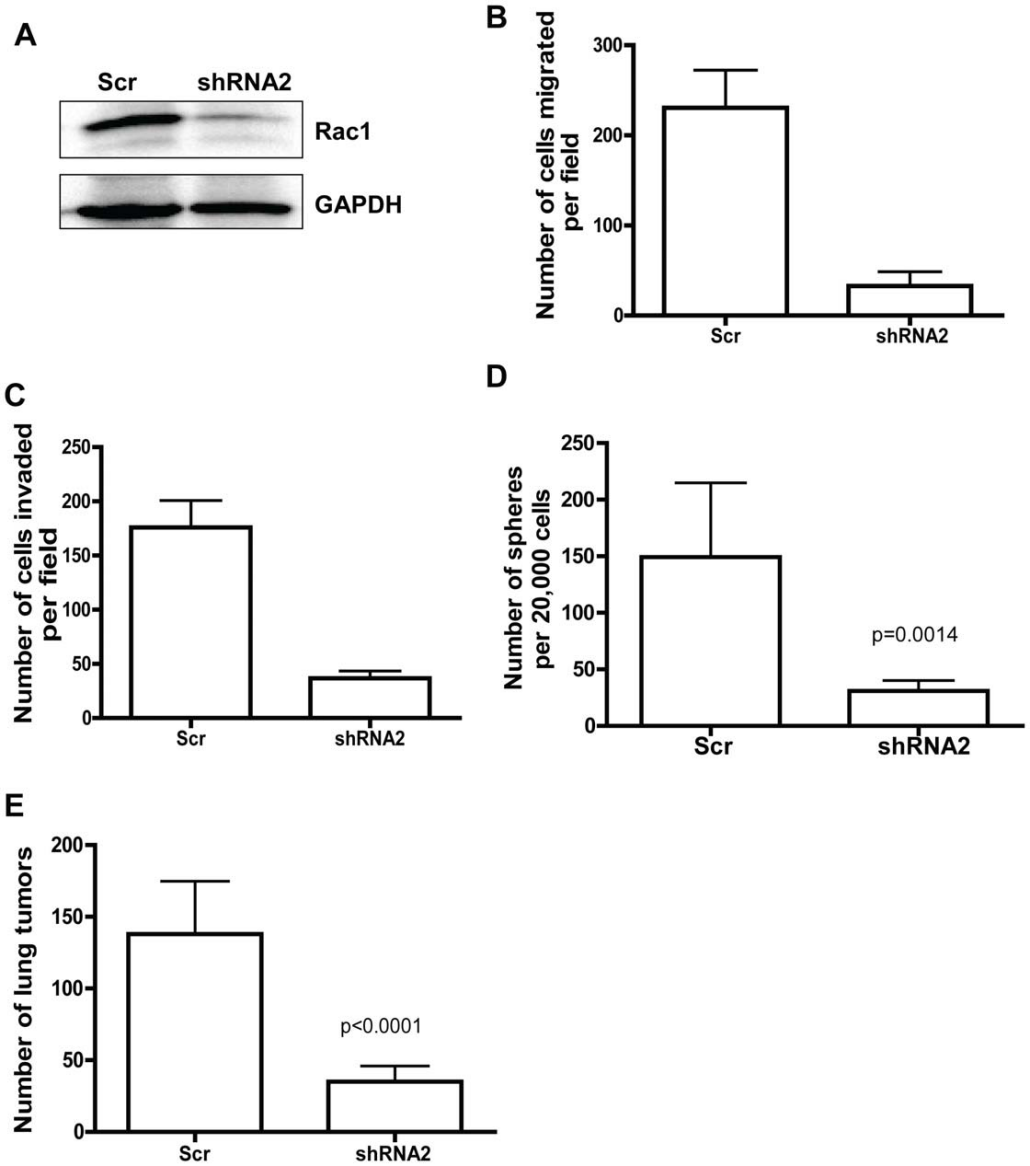
Rac1 targeting inhibits primary non-small cell lung cancer cell migration, invasion, sphere forming and lung colonization activities. (A) Cells isolated from primary tumor sample were infected with either scr or Rac1 shRNA and sorted cells were subjected to Rac1 western blot analysis. GAPDH was used as loading control. Above depicted is a representative of multiple western blots. (B, C, D) Primary human adenocarcinoma cells were plated for either trans-well migration assay (B) or invasion assay (C) or sphere assay (D). For sphere assay, sorted cells were directed plated in sphere growth media described in methods section. Number of spheres formed after 10 days were counted using light microscope. All the assays were performed in triplicates and error bar represents SD. Results are representative of three independent experiments. (E) For lung colonization assay, 5×10^5^ sorted cells were injected into tail vein of NSG mice (n = 4 per condition) and mice were sacked after 6 weeks. Number of lung tumors was counted under dissecting microscope. Error bar represents SD.

To further examine the relevance of Rac1 targeting on NSCLA cells, we have determined the functional outcomes of Rac1 knockdown in primary patient NSCLA cells that were TTF-1 positive (data not shown). Primary human tumor cells were infected with the lentivirus expressing Scr or Rac1 shRNA and subsequent Western blot analysis confirmed the effective knockdown of Rac1 protein expression in the isolated cells (Fig. 7A). Similar to our observations in NSCLA cell lines, Rac1 knockdown in the primary patient cells showed a decrease in trans-well migration and invasion activities (Fig. 7B, 7C). Significantly, the sphere formation capability was also compromised upon Rac1 knockdown (Fig. 7D, lung adenocarcinoma sample #1) or NSC23766 treatment in two different lung adenocarcinoma samples (Fig. S6, lung adenocarcinoma sample #2). Finally, Rac1 shRNA was effective in suppressing the primary tumor cell colonization in lung compared to Scr control (Fig. 7E). These results suggest that Rac1 targeting is beneficial to suppressing patient NSCLA cancer stem cell activity.

## Discussion

In the present work we show that Rac1 is required for adhesion, migration and lung colonization of NSCLA cells. SP cells isolated from human adenocarcinoma cell lines have enriched lung colonization activity in immunodeficient mice. We further determined that this is associated with their elevated Rac1 activity and increased migration and invasion, as well as increased anchorage-independent growth ability. Importantly, the lung colonization, migration, and invasion activities of both SP and non-SP cells can be effectively blocked by either a Rac1 inhibitor or Rac1 knockdown using shRNA, and the effect may be applicable to the CD133^+^ and CD133^−^ cell populations. These beneficial effects on tumor cell suppression also appear to apply to primary patient derived NSCLA cells and are irrespective of the p53 or K-ras mutation status (Table S1). We propose that Rac1 plays a crucial role in regulating CSC tumor initiating, metastatic activities and thus represents a novel and useful therapeutic target in NSCLA.

The discovery of a population of self renewing cancer stem cells in multiple types of cancer including lung cancer has led to the proposal that CSCs, not non-CSCs in a given tumor, are responsible for tumor initiation and possibly metastasis [12], [13], [24]. Clinical observations of secondary metastasis post-surgery or -chemotherapy has added to the concept that a residual population of tumor cells may escape conventional therapy and give rise to heterogeneous tumors at metastatic sites. Thus, CSCs are likely the source cells present in the primary tumors which possess unique proliferative and metastatic advantages. To date, CSCs are mostly identified by using various markers. Breast cancer cell lines enriched for CD44^+^/CD24^−^ markers express higher levels of pro-invasive genes and display higher invasive potential [25]. In pancreatic cancer, CD133^+^/CXCR4^+^ cells are shown to be responsible for metastasis [26]. In human liver cancer, CD45^−^/CD90^+^/CD44^+^ cells form metastatic lesions in the lungs of immune compromised mice, and blockage of CD44 activity by an antagonizing antibody is shown to block tumor growth and metastasis [27]. In line with these observations, SP lung cancer cells have been shown to possess enriched CSC activity by forming subcutaneous tumors in xenograft mice at a reduced cell number [12]. Here, we found that SP cells isolated from NSCLA cells display increased migration, invasion, homing and lung colonization activities, in addition to enriched tumorigenic capability. These observations are consistent with the notion that CSCs are unique in their metastatic potential as well as tumor initiation ability.

Conventional chemotherapeutic agents mainly target malignant cells by either inducing DNA damage or blocking DNA replication. CSCs may be resistant to the effect of these agents through their elevated drug resistance or relative quiescence [28]. To apply the CSC theory, several innovative therapeutic strategies aimed at eradicating CSCs have been developed. To tackle leukemia CSC, neutralizing antibodies to autocrine signaling mediators important for CSC growth such as CD123 [29], antagonist for leukemia CSC localization in the bone marrow niche such as CXCR4-inhibitor AMD-3100 and CD44 antibodies [30], and inhibitors of signaling pathways specifically upregulated in CSCs that are important for the self renewal such as NFκ-β inhibitor parthenolide [31], have been shown to have efficacy in AML or CML. In solid cancers, IL4 has been shown to be useful in colorectal cancer stem cell suppression [32], [33], BMP4 was found to induce glioblastoma CSCs to differentiate into non-CSCs [34], and salinomycin, a selective potassium ionophore, could target breast cancer stem cell proliferation and induce differentiation [15]. Our current study adds to this list of potential CSC targeting approaches by presenting evidence that Rac1 inhibition could be efficacious for suppressing both tumor initiation and metastasis of NSCLA CSCs.

Effective targeting of CSCs for therapeutic benefit requires accurate identification of the CSC population. In lung cancer, CD133^+^, ALDH^high^, and SP have been used as markers to track CSC activity, and *in vitro* anchorage-independent growth, sphere formation assays and subcutaneous xenograft models have been employed as readouts for the relative CSC activity [11], [12], [13]. Although each of these markers may help enrich the CSCs, it is clear that they do not accurately identify the tumor initiating cells in human NSCLA tumors, as this population might be quite complex and could be represented by an overlap of several different markers. Thus, using SP or CD133 as the sole marker for CSC identification may not include all CSC activity in a bulk culture. To this end, it will be interesting to determine whether SP cells overlap with CD133^+^ cells so that a more pure population of CSCs can be isolated.

The issue of CSC plasticity has been raised recently by several studies [16], [35]. It seems possible that both CSCs and non-CSCs could be dynamic populations – CSCs able to give rise to non-CSCs, and non-CSCs may convert to CSCs in given conditions. A recent review [36] raised an interesting point that the reprogramming from differentiating cancer cells to CSCs, unlike that of reprogramming of fully differentiated cells into iPS cells, might occur readily in cancer cells. Recent evidence from Boiko *et al* [35] and Roesch *et al* [16] have shown that non-CSCs can indeed covert to CSCs under suitable conditions. We have found that highly purified non-SP cells still retain residue CSC activity *in vitro* and in mice, raising the possibility that non-CSCs of NSCLA can produce CSCs or SP marker is insufficient in identifying CSCs. This consideration highlights the difficulty of utilizing the CSC theory to design new strategies against cancer, since it can be inferred that it will be necessary to effectively target both CSCs and non-CSCs or multiple marker populations in order to achieve true therapeutic benefits. Importantly, we show that targeting Rac1 can effectively block the lung metastatic and tumor initiating activities of both SP and non-SP NSCLA cells, and such benefits may apply to the CD133^+^/CD133^−^ tumor initiating populations. Future stringent evaluation how Rac1 contributes to various aspects of CSC activity in multiple marker positive subpopulations will significantly add to the understanding of Rac1 targeting in lung tumorigenesis.

## Supporting Information

Figure S1 **Effects of stable Rac1 suppression on H441, H1299 and H23 cell proliferation.** (A) Cell lysates collected from either scrambled shRNA (Scr) or Rac1 shRNA (shRNA1) H441 cells were subjected to Rac1 western blot analysis. GAPDH was used as loading control. (B) Sorted cells were plated and incubated with BrdU in log phase of cell growth. Cells are trypsinized and stained with BrdU antibody, 7AAD to perform cell cycle analysis. Assay was performed in triplicates and a representative experiment is shown. Error bars represent SD. (C, E) H1299 (C) and H23 cells (E) were transduced with lentivirus expressing either scr shRNA or Rac1 shRNA. 72 hours after the transduction, cells were plated for a proliferation assay. The number of cells was determined by MTS measurements at different time points. Assays were performed in triplicates and error bars represent SD. (D, F) The cells were plated for soft agar colony formation assay. Number of colonies formed was counted under light microscope after 2–3 weeks. Assays were performed in triplicates and error bar represents SD.
(TIF)

Figure S2 **Effect of Rac1 suppression on focal adhesion complexes, adhesion, migration and invasion.** (A) A549 cells expressing either scr or Rac1 shRNAs were sorted and plated. Cell lysates collected from adherent cells were processed for p-MLC, p-FAK, p-Paxillin, Rac1 Western blot analysis. GAPDH was probed as a loading control. Data is representative of three experiments. (B, C, D) H441 cells expressing either scr or Rac1 shRNAs were sorted and were plated on fibronectin coated plates for adhesion assay (B), on trans-well migration plate for migration assay (C) or matrigel coated invasion plates for invasion assay (D). All assays were performed in triplicates and data are representative of three independent experiments. Error bars represent SD. (E, F) H1299 (E) and H23 cells (F) were transduced with either scr shRNA or Rac1 shRNA and 72 hours later cells were plated for the trans-well migration assay. Cells migrated overnight were stained and counted. The assay was performed in triplicates and error bar represents SD.
(TIF)

Figure S3 **Suppression of Rac1 expression in H441 cells inhibits lung colonization in mice.** (A) Lung colonization assay was performed with scr or Rac1shRNA expressing H441 cells. Number of tumors formed after 12 weeks were counted and error bar represents SD. Data is representative of three independent experiments. (B) H441 cells expressing either scr or Rac1 shRNA were plated for sphere assay as described in Methods. Number of spheres formed after 10 days were counted under light microscope. Assay was performed in triplicates and error bars represent SD. Depicted is a representative of two independent experiments. (C) HBEC cells expressing either scr or Rac1 shRNA were plated for the sphere assay. Number of spheres formed after 10 days were counted under light microscope. The assay was performed in triplicates and error bars represent SD.
(TIF)

Figure S4 **Properties of H441 SP and non-SP cells under Rac1 suppression.** (A) Quantitative RT-PCR for ABCG2 transporter gene was performed using RNA collected from sorted cells. ABCG2 expression was normalized to GAPDH and the relative expression was represented as fold-change from control cells. RT-PCR experiment was performed in triplicates and depicted is a representative of four independent experiments. Error bars represents SD. (B) H441 SP cells were plated for migration assay either in the presence of a vehicle or a Rac inhibitor, NSC23766 (50 µM). Cells migrated overnight were quantified as described in Methods. Error bars represent SD and the depicted is representative of two independent experiments. (C) H441 SP cells were plated for invasion assays in the presence or absence of the Rac inhibitor NSC23766 (50 µM). Cells invading through matrigel were quantified as described in Methods. Error bars represent SD and the depicted is representative of two independent experiments. (D) SP, NSP, or non-sorted cells were plated in 96-well plates and the proliferation was measured by the MTS assay. Assays were performed in triplicates and error bars represents SD. Depicted is a representative of three independent experiments. (E) SP, non-SP, or non-sorted cells were plated for soft agar colony assay and the colonies were quantified after 2 weeks. Assays were performed in triplicates and error bar represents SD. Data is representative of two independent experiments. (F) H441 cells were stained with Heochst dye and analyzed by flow cytometry. Depicted is representative of three independent analyses. (G) SP or NSP H441 cells (3×10^4^) were sorted and injected into tail vein of NSG mice (n = 3 per condition). Lungs were collected after 10–12 weeks and number of tumors formed in each lung was counted. Error bar represents SD. Depicted is representative of three independent experiments. (H) H441 SP, NSP, or non-sorted cells were plated for soft agar assay and colonies formed after 21 days were counted. Assays were performed in triplicates and error bar represents SD. Data is representative of two independent experiments.
(TIF)

Figure S5 **Effect of Rac1 suppression on cell actin organization.** (A) Cell lysates collected from FACS sorted A549 cells were subjected to Rac1 western blot analysis. GAPDH was used as loading control. Shown is a representative of three Western blots. (B) FACS sorted A549 cells were plated onto fibronectin coated slides and stained for actin cytoskeleton and nuclei by rhodamine-phalloidin and DAPI, respectively. Shown is a representative of several images obtained in two independent experiments.
(TIF)

Figure S6 **Effect of Rac1 inhibition on sphere formation.** Cells isolated from primary human lung adenocarcinoma were plated for sphere assay either in the presence of vehicle or NSC23766 (50 µM). The number of spheres formed after 10 days were counted under microscope. Assays were performed in triplicates and error bars represent SD. Depicted is a representative of two independent experiments.
(TIF)

Table S1 **Summary of Rac1 knockdown data in NSCLA cell lines.**
(TIF)
