# Supplementary figures and images for: Rac1 Targeting Suppresses Human Non-Small Cell Lung Adenocarcinoma Cancer Stem Cell Activity
Source: PLoS One. 2011 Feb 9;6(2):e16951. doi: 10.1371/journal.pone.0016951 (PMC3036726; doi:10.1371/journal.pone.0016951)

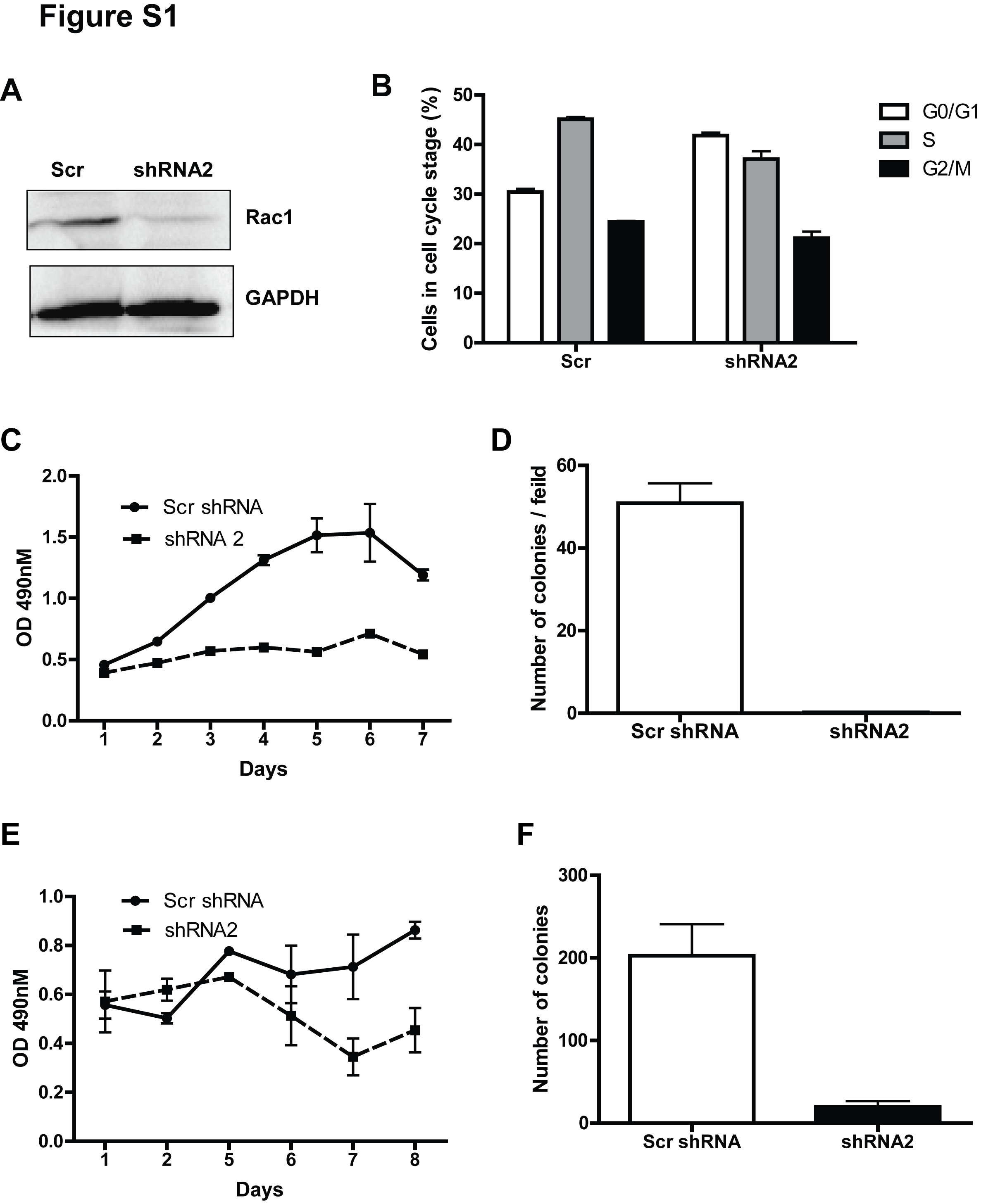

Supplement: Figure S1 — Effects of stable Rac1 suppression on H441, H1299 and H23 cell proliferation. (A) Cell lysates collected from either scrambled shRNA (Scr) or Rac1 shRNA (shRNA1) H441 cells were subjected to Rac1 western blot analysis. GAPDH was used as loading control. (B) Sorted cells were plated and incubated with BrdU in log phase of cell growth. Cells are trypsinized and stained with BrdU antibody, 7AAD to perform cell cycle analysis. Assay was performed in triplicates and a representative experiment is shown. Error bars represent SD. (C, E) H1299 (C) and H23 cells (E) were transduced with lentivirus expressing either scr shRNA or Rac1 shRNA. 72 hours after the transduction, cells were plated for a proliferation assay. The number of cells was determined by MTS measurements at different time points. Assays were performed in triplicates and error bars represent SD. (D, F) The cells were plated for soft agar colony formation assay. Number of colonies formed was counted under light microscope after 2–3 weeks. Assays were performed in triplicates and error bar represents SD. (TIF) [file pone.0016951.s001.tif]

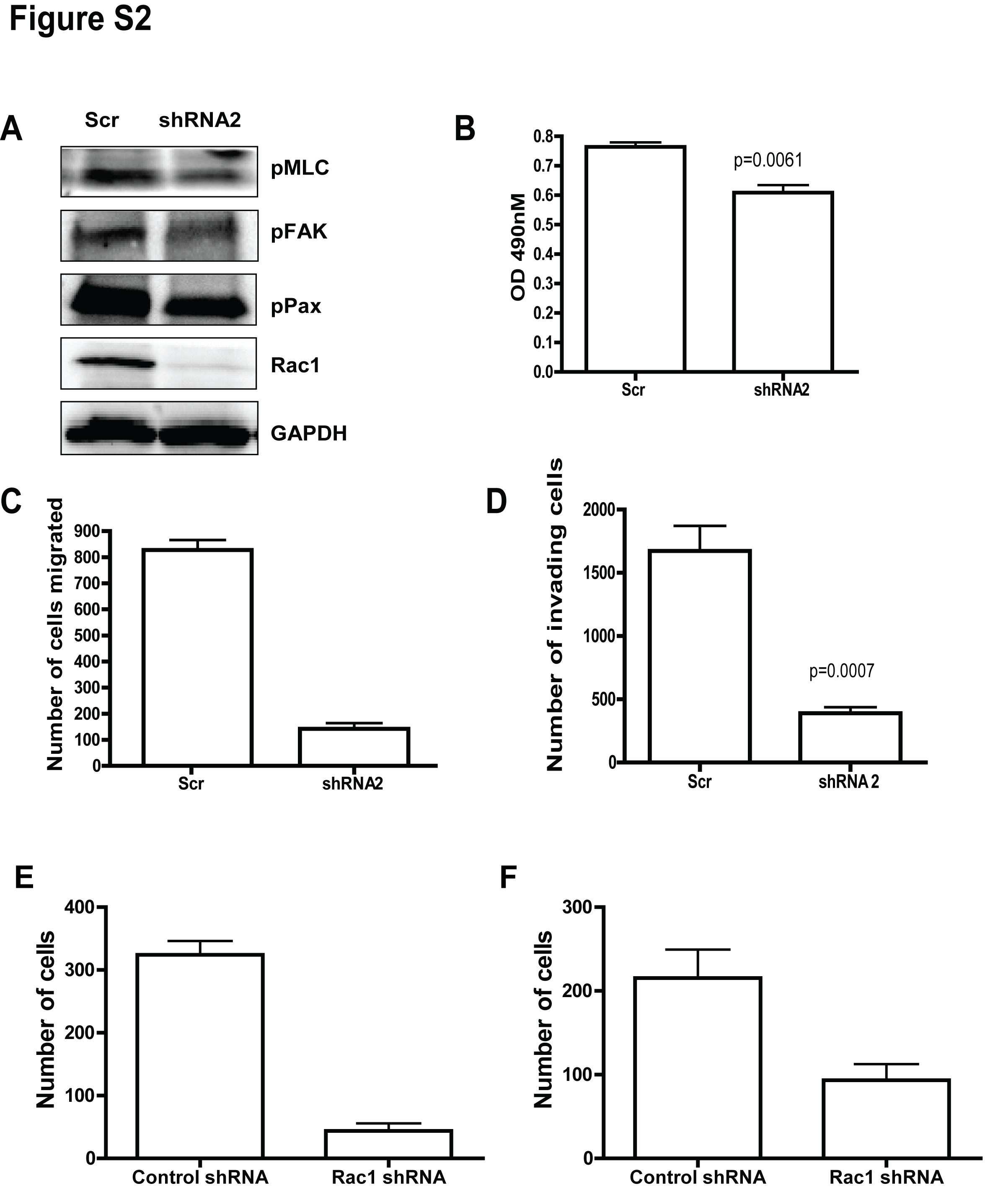

Supplement: Figure S2 — Effect of Rac1 suppression on focal adhesion complexes, adhesion, migration and invasion. (A) A549 cells expressing either scr or Rac1 shRNAs were sorted and plated. Cell lysates collected from adherent cells were processed for p-MLC, p-FAK, p-Paxillin, Rac1 Western blot analysis. GAPDH was probed as a loading control. Data is representative of three experiments. (B, C, D) H441 cells expressing either scr or Rac1 shRNAs were sorted and were plated on fibronectin coated plates for adhesion assay (B), on trans-well migration plate for migration assay (C) or matrigel coated invasion plates for invasion assay (D). All assays were performed in triplicates and data are representative of three independent experiments. Error bars represent SD. (E, F) H1299 (E) and H23 cells (F) were transduced with either scr shRNA or Rac1 shRNA and 72 hours later cells were plated for the trans-well migration assay. Cells migrated overnight were stained and counted. The assay was performed in triplicates and error bar represents SD. (TIF) [file pone.0016951.s002.tif]

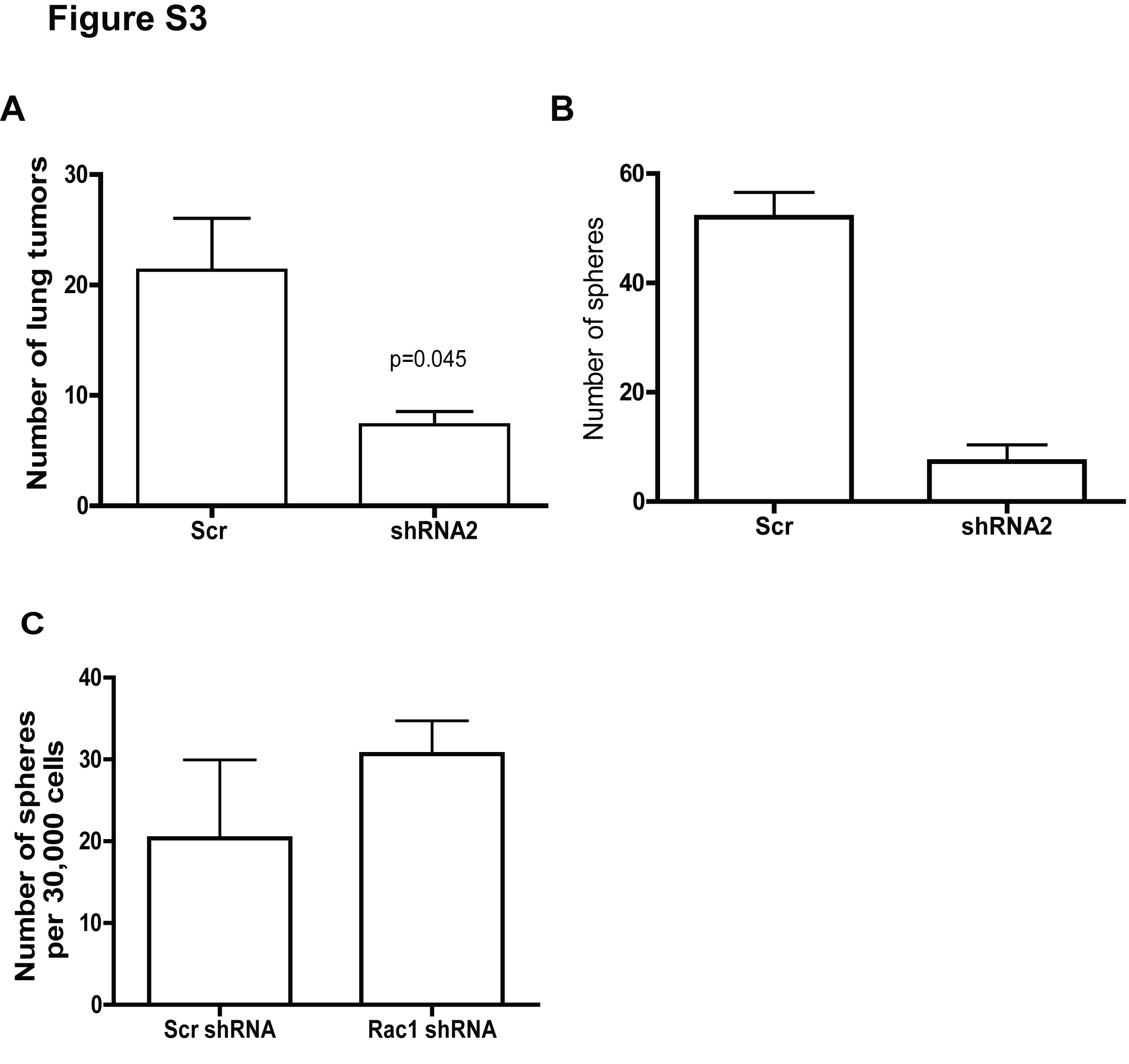

Supplement: Figure S3 — Suppression of Rac1 expression in H441 cells inhibits lung colonization in mice. (A) Lung colonization assay was performed with scr or Rac1shRNA expressing H441 cells. Number of tumors formed after 12 weeks were counted and error bar represents SD. Data is representative of three independent experiments. (B) H441 cells expressing either scr or Rac1 shRNA were plated for sphere assay as described in Methods. Number of spheres formed after 10 days were counted under light microscope. Assay was performed in triplicates and error bars represent SD. Depicted is a representative of two independent experiments. (C) HBEC cells expressing either scr or Rac1 shRNA were plated for the sphere assay. Number of spheres formed after 10 days were counted under light microscope. The assay was performed in triplicates and error bars represent SD. (TIF) [file pone.0016951.s003.tif]

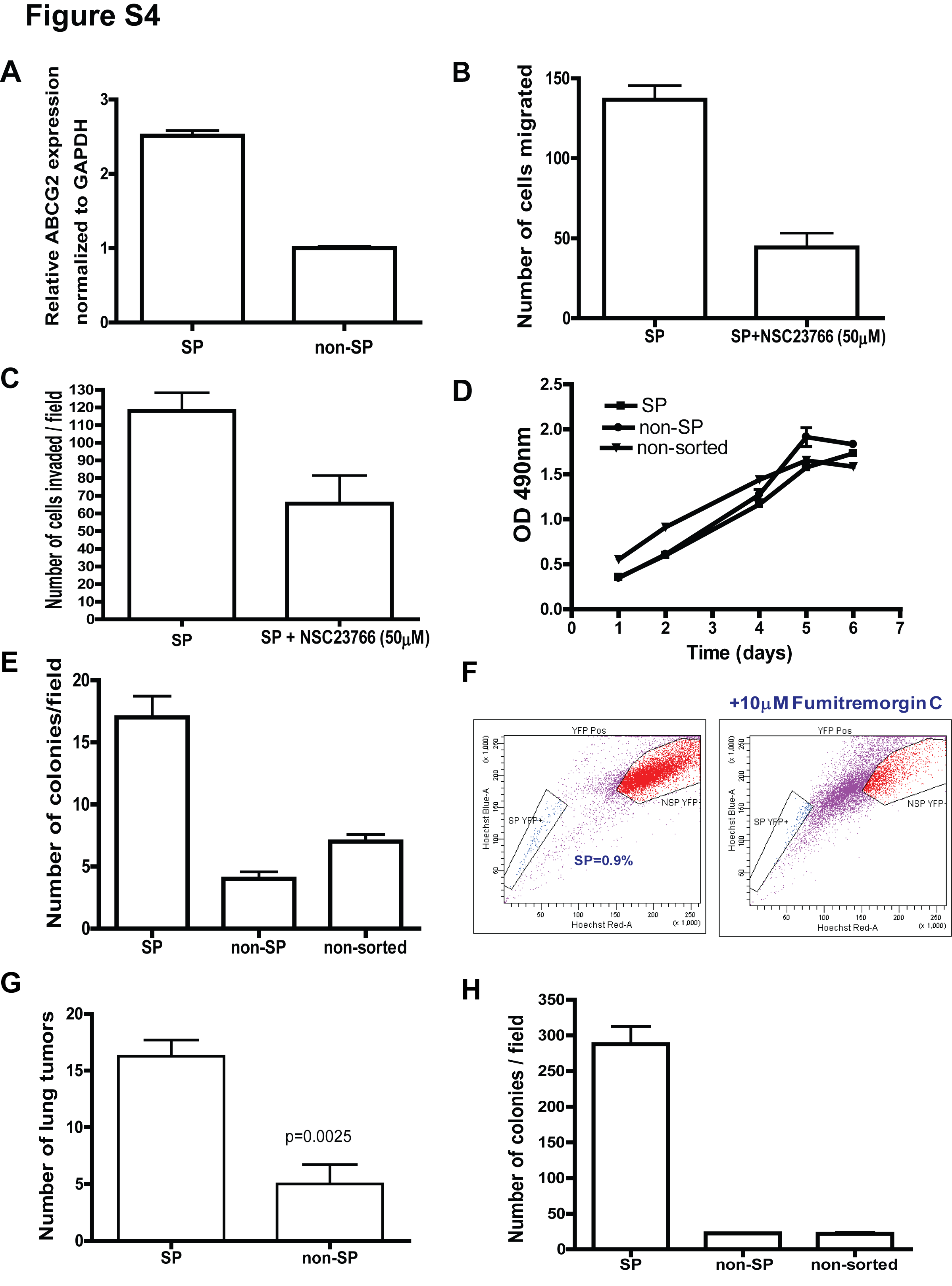

Supplement: Figure S4 — Properties of H441 SP and non-SP cells under Rac1 suppression. (A) Quantitative RT-PCR for ABCG2 transporter gene was performed using RNA collected from sorted cells. ABCG2 expression was normalized to GAPDH and the relative expression was represented as fold-change from control cells. RT-PCR experiment was performed in triplicates and depicted is a representative of four independent experiments. Error bars represents SD. (B) H441 SP cells were plated for migration assay either in the presence of a vehicle or a Rac inhibitor, NSC23766 (50 µM). Cells migrated overnight were quantified as described in Methods. Error bars represent SD and the depicted is representative of two independent experiments. (C) H441 SP cells were plated for invasion assays in the presence or absence of the Rac inhibitor NSC23766 (50 µM). Cells invading through matrigel were quantified as described in Methods. Error bars represent SD and the depicted is representative of two independent experiments. (D) SP, NSP, or non-sorted cells were plated in 96-well plates and the proliferation was measured by the MTS assay. Assays were performed in triplicates and error bars represents SD. Depicted is a representative of three independent experiments. (E) SP, non-SP, or non-sorted cells were plated for soft agar colony assay and the colonies were quantified after 2 weeks. Assays were performed in triplicates and error bar represents SD. Data is representative of two independent experiments. (F) H441 cells were stained with Heochst dye and analyzed by flow cytometry. Depicted is representative of three independent analyses. (G) SP or NSP H441 cells (3×10^4^) were sorted and injected into tail vein of NSG mice (n = 3 per condition). Lungs were collected after 10–12 weeks and number of tumors formed in each lung was counted. Error bar represents SD. Depicted is representative of three independent experiments. (H) H441 SP, NSP, or non-sorted cells were plated for soft agar assay and colonies formed after 21 da [file pone.0016951.s004.tif]

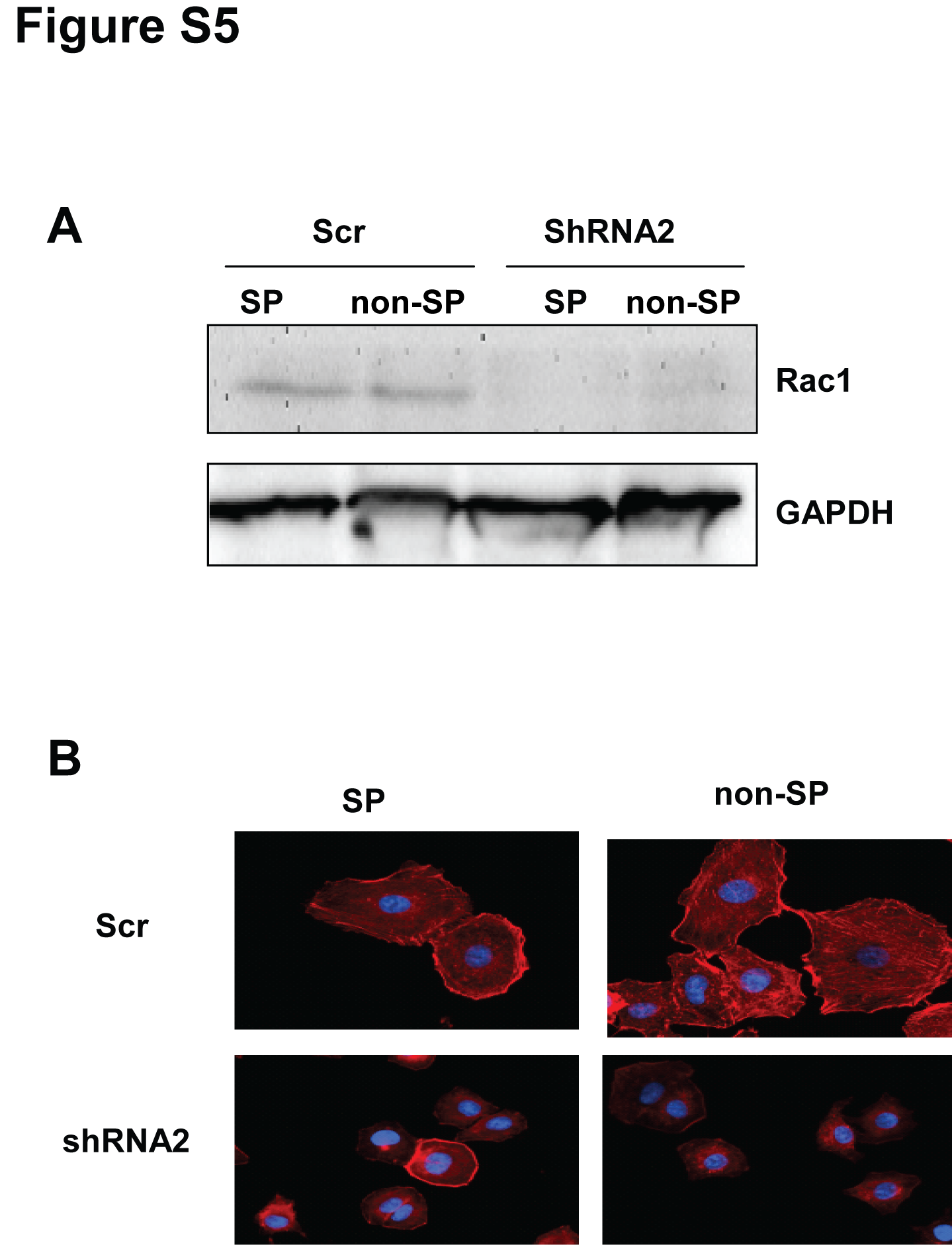

Supplement: Figure S5 — Effect of Rac1 suppression on cell actin organization. (A) Cell lysates collected from FACS sorted A549 cells were subjected to Rac1 western blot analysis. GAPDH was used as loading control. Shown is a representative of three Western blots. (B) FACS sorted A549 cells were plated onto fibronectin coated slides and stained for actin cytoskeleton and nuclei by rhodamine-phalloidin and DAPI, respectively. Shown is a representative of several images obtained in two independent experiments. (TIF) [file pone.0016951.s005.tif]

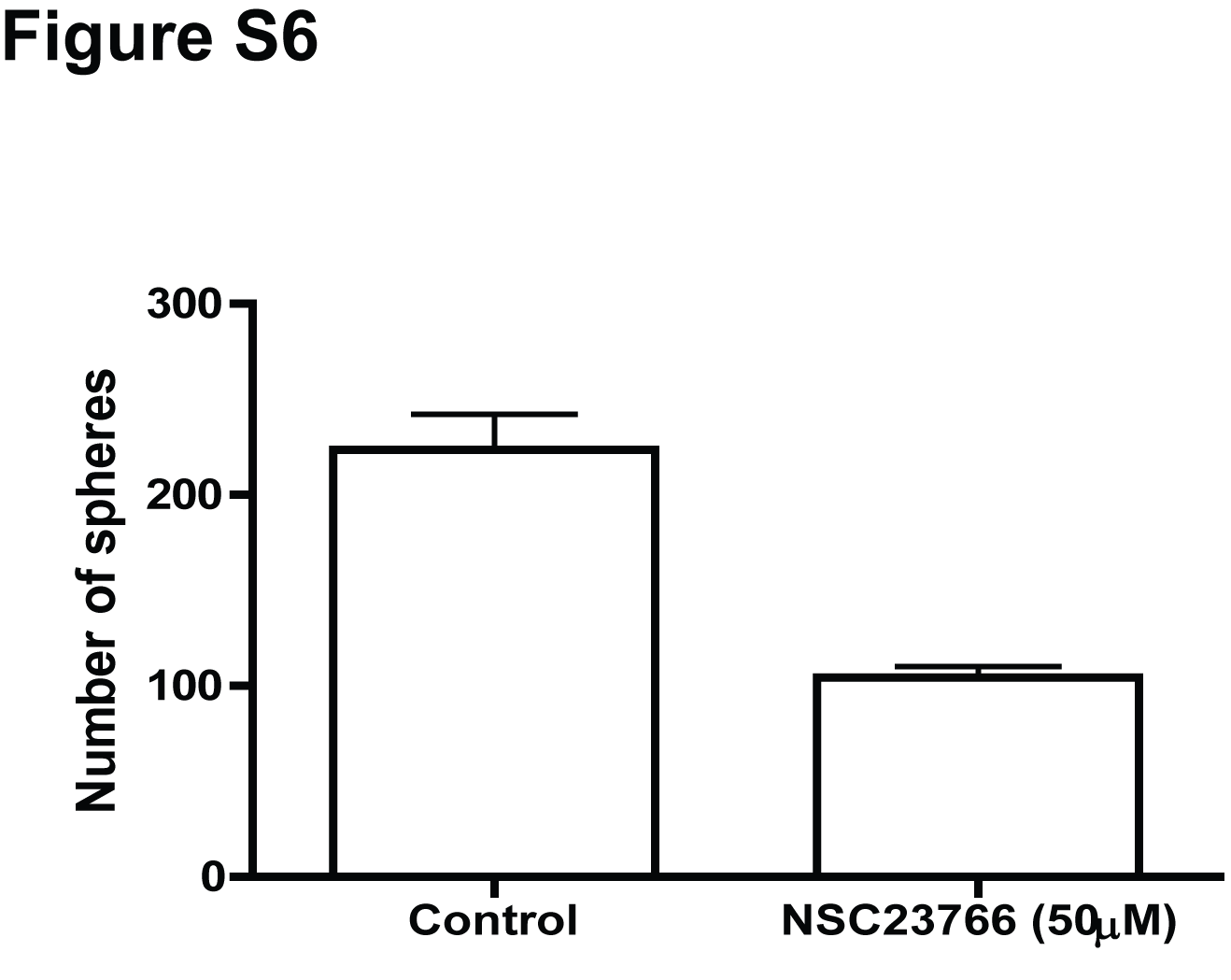

Supplement: Figure S6 — Effect of Rac1 inhibition on sphere formation. Cells isolated from primary human lung adenocarcinoma were plated for sphere assay either in the presence of vehicle or NSC23766 (50 µM). The number of spheres formed after 10 days were counted under microscope. Assays were performed in triplicates and error bars represent SD. Depicted is a representative of two independent experiments. (TIF) [file pone.0016951.s006.tif]

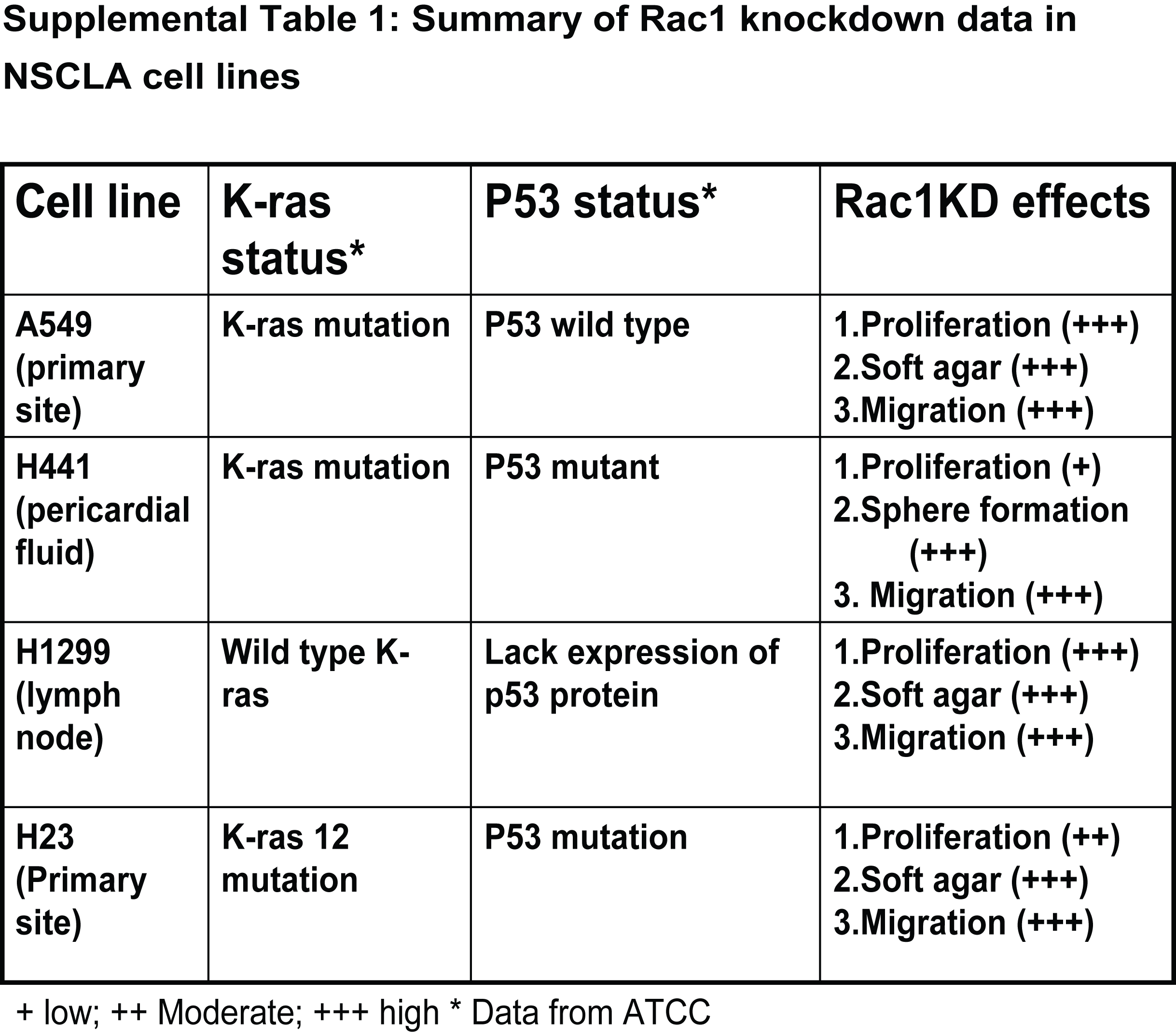

Supplement: Table S1 — Summary of Rac1 knockdown data in NSCLA cell lines. (TIF) [file pone.0016951.s007.tif]
